# Antibacterial Resistance Patterns Among Common Infections in a Tertiary Care Hospital in Saudi Arabia

**DOI:** 10.7759/cureus.31695

**Published:** 2022-11-20

**Authors:** Alqassem Y Hakami, Lujain H Felemban, Noor A Aljifri, Ghayda M Alyamani, Khadijah A Abosallamh, Rahaf A Aljohani, Taghreed Aldosary, Abdulmajeed Basheikh

**Affiliations:** 1 College of Medicine, King Saud Bin Abdulaziz University for Health Sciences, Jeddah, SAU; 2 Research Office, King Abdullah International Medical Research Center, Jeddah, SAU; 3 Deaprtment of Medical Sciences-Oral Biology, Ministry of National Guard-Health Affairs, King Abdulaziz Medical City, Jeddah, SAU; 4 Department of Family Medicine, Ministry of National Guard-Health Affairs, King Abdulaziz Medical City, Jeddah, SAU

**Keywords:** klebsiella pneumoniae, pseudomonas aeruginosa, antibiotic-resistant bacteria, escherichia coli, e.coli, urinary tract infection, respiratory tract infection, resistance, antibiotics

## Abstract

Background

The rapid emergence of antibiotic-resistant bacteria threatens the control of infectious diseases by reducing treatment effectiveness, prolonging illness duration, and increasing healthcare costs. This study aimed to identify the common rate of bacterial resistance against antibacterial agents in tertiary healthcare providers in Saudi Arabia.

Methodology

This retrospective cross-sectional observational study was conducted from May 2016 to December 2019 on 1,151 urinary tract infection (UTI) and respiratory tract infection (RTI) positive cultures collected from participants aged 15 years or older who received antibiotic treatment. The obtained variables included age, gender, diagnosis, antibiotic type, specimen source, culture results, and sensitivity test results.

Results

The most common bacteria in UTI were *Escherichia* *coli* (46.7%), followed by *Klebsiella* *pneumoniae* (30.5%). Moreover, *E.* *coli* was most resistant to ampicillin (56.4%), followed by ceftriaxone (33.8%). Among the respiratory cultures, the most frequently isolated pathogen was *Pseudomonas aeruginosa* (28.5%), followed by* K*. *pneumoniae* (17.6%). The 162 respiratory *P*. *aeruginosa* isolates were most resistant to piperacillin/tazobactam (51.9%), followed by ciprofloxacin (25%) and ampicillin (10.6%).

Conclusion

High levels of antibiotic resistance were observed in both Gram-negative and Gram-positive bacteria. This indicates a need for better implementation of antibacterial stewardship and increased awareness of appropriate antibiotic use to limit the rapid spread of antibacterial resistance.

## Introduction

The introduction of antibiotics to the medical field was one of the greatest discoveries in the history of medicine. When penicillin was introduced in the 1940s by Alexander Fleming, a new era of therapeutic medicine was established [1]. The outcomes of bacterial infections saw a great turnaround as fatal and severe infections became easily treatable. However, the efficiency of antibiotics has decreased as many available antibiotics are no longer effective along with the emergence of antibiotic-resistant (ABR) strains. Importantly, antibiotic resistance discovery is related to resistance detection in clinical samples; however, the resistance might be discovered earlier according to the observation from laboratory samples.

Globally, it is estimated that ABR infections are responsible for approximately 700,000 deaths per year [2,3]. If no preventative actions are taken, it is predicted that infections caused by ABR bacteria will have a mortality rate exceeding that of cancer and become the most common cause of death by the end of 2050 [2,3]. According to the Centers for Disease Control and Prevention (CDC), approximately 35,900 deaths out of 2,868,700 ABR cases are expected to be reported annually in the United States [2]. In 2012, Aly et al. investigated antimicrobial resistance in 37,295 bacterial isolates collected from different hospitals in the Gulf region. Within this sample, the most prevalent microorganism was *Escherichia coli*, followed by *Klebsiella pneumoniae*, *Pseudomonas aeruginosa*, methicillin-resistant *Staphylococcus aureus* (MRSA), *Acinetobacter*,* Clostridium difficile*, and *Enterococcus* [4]. In addition, a study conducted by the Saudi national surveillance on Gram-positive cocci revealed that 32% of *S. aureus* belonged to MRSA, 33% of *S. pneumoniae* were resistant to penicillin G, and 26% of *S. pneumoniae* were resistant to erythromycin [3]. In the western region of Saudi Arabia, Alam et al. reported bacterial resistance to trimethoprim/sulfamethoxazole (48.6%), ampicillin (49.3%), piperacillin (59.3%), and methicillin (50.3%) [5].

Bacteria have the unique ability to lower or eliminate the antimicrobial efficacy of drugs and chemical agents [6]. This may occur through natural resistance (e.g., β-lactamase production) or acquired resistance [7,8]. Acquired bacterial resistance may occur through four mechanisms. One of these mechanisms is the production of enzymes that modify or inhibit antibiotic action [7-9]. Another mechanism is through changes in the permeability of bacterial cell walls [7]. Bacteria can also acquire resistance through disruptions in protein synthesis [7], alterations in metabolic pathways, or genetic mutations [8]. Finally, bacteria can acquire resistance from the transferred copy of the plasmid (R-plasmid genes) of a previously resistant bacteria [7-9].

The development of antibiotic resistance appears to be inevitable [10]. However, the overuse and misuse of antibiotics are accelerating this process [11]. The misuse of antibiotics is a complex problem driven by several factors related to patients, healthcare providers, and institutional healthcare regulations [12]. Public knowledge, awareness, and attitudes regarding antibiotic use are strong determinants of antibiotic misuse [13]. In a systematic review conducted by Alhomoud et al.in 2017 and* *demonstrated the use of antibiotics in the Middle East, the overall prevalence of participants who used antibiotics as self-prescription ranged from 19% to 82% [14]. The highest prevalence of self-prescription antibiotics was reported in Yemen and Oman followed by Saudi Arabia [14]. Access to antibiotics without a prescription and gaps in knowledge and safe practices regarding antibiotics' use (e.g., keeping leftover antibiotics from an uncompleted course for future use and sharing antibiotics with others) were among the reported reasons for self-medication with antibiotics [14]. Furthermore, prescribers’ knowledge and attitudes regarding antibiotic use and resistance have been reported to determine the quality of antibiotic prescriptions [15]. One core problem underlying improper antibiotic prescription is the lack of sufficient diagnostic tests to rapidly identify pathogens and their antibiotic susceptibility profiles [16]. Another proven risk factor for antibiotic resistance is travel, specifically during the Hajj season, when the acquisition and transmission of infectious diseases (including those caused by ABR bacteria) are common occurrences [17].

The topic of antibiotic resistance has been approached from many perspectives for a wide variety of clinical and social practices and implications. However, the present research specifically aimed to assess the prevalence of ABR infections in Ministry of National Guard-Health Affairs (MNGHA), Jeddah, Saudi Arabia. In a study conducted in the western region of Saudi Arabia, pneumonia was the most prevalent infectious disease reported in patients aged 26-45 years [18]. Additionally, pneumonia and urinary tract infections (UTIs) were the most prevalent forms of infectious diseases among female patients [18]. In the central region of Saudi Arabia, respiratory tract infections (RTIs) and UTIs have been found to be the most frequent complaints encountered in emergency departments [19]. The availability of updated epidemiological data from a given region or community is important not only for the optimization of empirical therapies but also for the implementation of an effective antimicrobial stewardship program in hospitals [20].

## Materials and methods

Selection criteria

An observational cross-sectional quantitative study (with non-probability convenience sampling) was conducted in the MNGHA, Jeddah, Saudi Arabia. For this study, patients were selected according to the following criteria: male and female Saudi inpatients and outpatients aged 15 years or older who had received antibiotic treatments prior to the initiation of the study for UTIs and/or RTIs. This sample excluded the oncology department, patients infected with tuberculosis (TB), and patients infected with human immunodeficiency virus (HIV).

Sample size calculation

The sample size was calculated using Raosoft® software (Raosoft Inc., Seattle, United States). Approximately 231,000 patients received antibiotic treatments in MNGHA, Jeddah, between May 2016 and December 2019. At a 95% confidence level, an estimated 59.1% prevalence of ABR patients, and a 5% margin of error, the required minimum sample size was estimated at 371 samples. All patients who met the sample criteria from May 2016 to December 2019 were included in the study.

Data were obtained from the BESTCare system (ezCaretech, Torrance, California, United States) using a data collection sheet. The collected numerical variables included age and date of diagnosis, and the collected categorical/nominal variables included gender, hospital setting, diagnosis, antibiotic type, specimen source, culture results, and sensitivity test results.

Data analysis

Parametric and non-parametric approaches were used to describe the numerical data (age and date of diagnosis). Percentages were used to describe the categorical variables (gender, hospital setting, diagnosis, antibiotic type, specimen source, type of organism, and sensitivity test results). Chi-square or Fisher exact test was used to compare categorical data, while t-test and ANOVA were used to make comparisons between categorical and numerical variables. A p-value of less than 0.05 was statistically significant. All data were analyzed using IBM SPSS Statistics for Windows, Version 20.0 (Released 2011; IBM Corp., Armonk, New York, United States).

Ethical approval

The study was carried out in line with the Helsinki protocol and ethical approval from the Institutional Review Board of King Abdullah International Medical Research Centre, Jeddah, Saudi Arabia, was duly acquired prior to conducting this study (approval number: SP20/050/J, dated April 22, 2020). No names and Identities (IDs) were collected from the participants, and the data were stored within 64‑bit encrypted software on the work computer of the primary investigator that was not liable to be breached by nonauthorized persons.

## Results

A total of 1,151 isolates were obtained from the BESTCare system in MNGHA, Jeddah, Saudi Arabia, between May 2016 and December 2019. These samples were categorized into age groups. Overall, 52.7% (n = 607) of these samples were collected from female patients, and 78.2% (n = 900) and 21.8% (n = 251) were collected from inpatients and outpatients, respectively. Data regarding patient demographics, hospital settings, and specimen types are displayed in Table 1.

**Table 1:** Participants’ demographic data.

		n=1151	%
Age			
	15–25 years	55	4.8
	26–35 years	40	3.5
	36–45 years	129	11.2
	46–55 years	107	9.3
	56–65 years	175	15.2
	66–75 years	232	20.2
	76–85 years	294	25.5
	86–95 years	101	8.8
	>95 years	18	1.6
Gender			
	Male	544	47.3
	Female	607	52.7
Status			
	Outpatient	251	21.8
	Inpatient	900	78.2
Diagnosis			
	Respiratory infection	568	49.3
	Urinary tract infection	583	50.7
Date of diagnosis			
	2016	174	15.1
	2017	352	30.6
	2018	371	32.2
	2019	254	22.1

Regarding specimen sources, 49.3% (n = 568) of the samples were obtained from respiratory specimens, 50.7% (n = 583) were obtained from urine specimens, and the sources of seven specimens were not documented; thus, the total number of specimens was 1,144 (Table 2). 

**Table 2:** Specimen source. MRSA: methicillin-resistant *Staphylococcus aureus*

		n=1144	%
Specimen source			
	Urine	578	50.5
	Sputum	397	34.7
	Endotracheal aspiration	62	5.4
	Tracheal aspiration	51	4.5
	Nasal swab	21	1.8
	Urinary catheter	13	1.1
	MRSA culture	10	.9
	Bronchoalveolar lavage	5	.4
	Nasopharyngeal airway (NPA)	3	.3
	Bronchial biopsy	1	.1
	Bronchial wash	1	.1
	Pleural fluid	1	.1
	Tissue culture	1	.1

**Figure 1:**
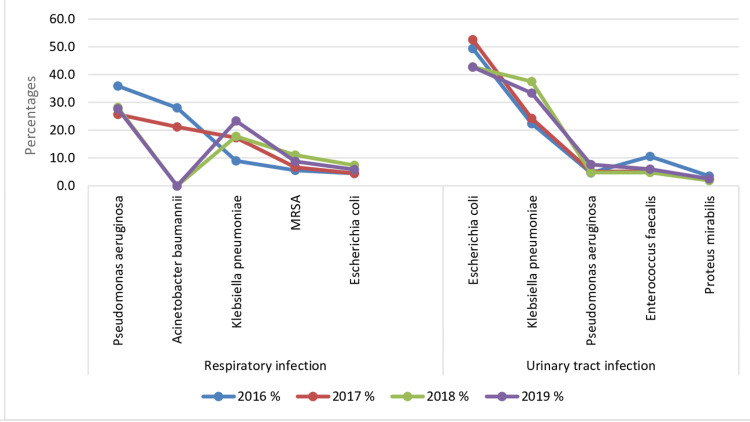
Bacterial composition: comparison between common bacterial isolates in urinary tract infections (UTIs) and respiratory tract infections (RTIs) during the study period. In ascending order, this figure lists the bacterial isolates (x-axis) most commonly found in UTIs and RTIs with their percentages (y‑axis) in each type of infection. The line graph represents changes in these bacterial isolates that occurred in 2016 (blue line), 2017 (red line), 2018 (green line), and 2019 (purple line). The percentages in RTIs were as follows: *Pseudomonas aeruginosa* 36% (2016), 25.7% (2017), 28.2% (2018), and 27.7% (2019); *Acinetobacter baumannii *28.1% (2016), 21.2% (2017), and 0% (2018 and 2019); *Klebsiella pneumoniae* 9% (2016), 17.3% (2017), 17.8% (2018), and 23.4% (2019); methicillin-resistant *Staphylococcus aureus* (MRSA) 5.6% (2016), 6.7% (2017), 11% (2018), and 8.8% (2019); *Escherichia coli *4.5% (2016 and 2017), 7.4% (2018), and 5.8% (2019). The percentages in UTIs were as follows: *E. coli* 49.4% (2016), 52.6% (2017), 42.8% (2018), and 42.7% (2019); *K. pneumoniae* 22.4% (2016), 24.3% (2017), 37.5% (2018), and 33.3% (2019); *P. aeruginosa* 4.7% (2016), 5.2% (2017), 4.8% (2018), and 7.7% (2019); *Enterococcus faecalis* 10.6% (2016), 5.2% (2017), 4.8% (2018), and 6% (2019); *Proteus mirabilis* 3.5% (2016), 2.3% (2017), 1.9% (2018), and 2.6% (2019).

The top 10 most common causative agents of UTIs and RTIs were *E. coli *(26.4%; n = 304), *K. pneumoniae* (24.2%; n = 278), *P. aeruginosa* (16.9%; n = 194), *Acinetobacter baumannii *(8.4%; n = 97), MRSA (3.6%; n = 42), *Enterococcus faecalis* (3%; n = 35), *S. aureus* (2.9%; n = 33), *Proteus mirabilis* (2.1%; n = 24), *Haemophilus influenzae* (2%; n = 23), and *S. pneumoniae* (1.8%; n = 21) (Figure 1, Table 3).

**Table 3:** Causative agents of urinary tract infections (UTIs) and respiratory tract infections (RTIs).

	Bacteria	n=1151	%
	Escherichia coli	304	26.4
	Klebsiella pneumoniae	278	24.2
	Pseudomonas aeruginosa	194	16.9
	Acinetobacter baumannii	97	8.4
	Methicillin-resistant *Staphylococcus aureus*	42	3.6
	Enterococcus faecalis	35	3.0
	Staphylococcus aureus	33	2.9
	Proteus mirabilis	24	2.1
	Haemophilus influenzae	23	2.0
	Streptococcus pneumoniae	21	1.8
	Serratia marcescens	18	1.6
	Stenotrophomonas maltophilia	16	1.4
	Enterobacter cloacae	9	.8
	Citrobacter koseri	8	.7
	Providencia stuartii	7	.6
	Enterobacter aerogenes	7	.6
	Enterococcus faecium	5	.4
	Burkholderia cepacia	5	.4
	Klebsiella oxytoca	4	.3
	Citrobacter freundii	2	.2
	Salmonella	2	.2
	Elizabethkingia meningoseptica	2	.2
	Alcaligenes faecalis	2	.2
	Serratia liquefaciens	1	.1
	Bacillus anthracis	1	.1
	Moraxella catarrhalis	1	.1
	Serratia fonticola	1	.1
	Morganella morganii	1	.1
	Staphylococcus capitis	1	.1
	Cedecea lapagei	1	.1
	Pseudomonas putida	1	.1
	Providencia rettgeri	1	.1
	Achromobacter xylosoxidans	1	.1
	Cronobacter sakazakii	1	.1
	Haemophilus parainfluenzae	1	.1
	*Pantoea *species	1	.1

The most common microbial causative agent of UTIs was *E. coli* (46.7%; n = 272), followed by *K. pneumoniae *(30.5%; n = 178), *E. faecalis* (6%; n = 35), *P. aeruginosa* (5.5%; n = 32), and *P. mirabilis* (2.2%; n = 13) (Table 4).

**Table 4:** Bacteria isolated from urine specimen sources.

Bacteria		n=583	%
	Escherichia coli	272	46.7
	Klebsiella pneumoniae	178	30.5
	Enterococcus faecalis	35	6.0
	Pseudomonas aeruginosa	32	5.5
	Proteus mirabilis	13	2.2
	Acinetobacter baumannii	11	1.9
	Citrobacter koseri	6	1.0
	Enterobacter cloacae	5	.9
	Enterococcus faecium	5	.9
	Providencia stuartii	5	.9
	Enterobacter aerogenes	4	.7
	Serratia marcescens	3	.5
	Staphylococcus aureus	2	.3
	Stenotrophomonas maltophilia	2	.3
	Salmonella	2	.3
	Methicillin-resistant *Staphylococcus aureus*	1	.2
	Streptococcus pneumoniae	1	.2
	Haemophilus influenzae	1	.2
	Serratia fonticola	1	.2
	Citrobacter freundii	1	.2
	Cedecea lapagei	1	.2
	Pseudomonas putida	1	.2
	*Pantoea *species	1	.2

Furthermore, *E. coli* was most resistant to ampicillin (56.4%), followed by ceftriaxone (33.8%), ciprofloxacin (3.8%), amoxicillin (2.6%), and trimethoprim/sulfamethoxazole (1.7%; p = 0.014). Similarly, *K. pneumoniae *was most resistant to ampicillin (69.7%), followed by ceftriaxone (23.9%), amoxicillin/clavulanate (2.8%), amoxicillin (1.7%), and ciprofloxacin (0.6%; p < 0.001). Meanwhile, *E. faecalis* was most resistant to ciprofloxacin (28.6%), followed by ampicillin (21.4%), erythromycin and clindamycin (14.3%), and vancomycin (7.1%; p = 0.619). The* P. aeruginosa* isolates were most resistant to piperacillin/tazobactam (53.8%), followed by ciprofloxacin and ampicillin/sulbactam (15.4%) and cefazolin and trimethoprim/sulfamethoxazole (7.7%; p = 0.023). Finally, *P. mirabilis *was most resistant to ampicillin (53.8%), followed by ciprofloxacin (23.1%), nitrofurantoin (15.4%), and trimethoprim/sulfamethoxazole (7.7%; p = 0.860). The complete results are illustrated in Table 5 and Table 6. 

**Table 5:** Resistance rate changes over the study period among urinary tract bacteria.

	Urinary tract infection bacteira (Resistant)	Year of diagnosis								p-value
		2016		2017		2018		2019		
		n=31	%	n=81	%	n=83	%	n=39	%	
Escherichia coli										0.550
	Ciprofloxacin	0	0.0	3	3.7	4	4.8	2	5.1	
	Ceftriaxone	13	41.9	30	37.0	23	27.7	13	33.3	
	Piperacillin/tazobactam	0	0.0	0	0.0	1	1.2	0	0.0	
	Amoxicillin-clavulanate	0	0.0	0	0.0	0	0.0	1	2.6	
	Cefazolin	0	0.0	1	1.2	0	0.0	0	0.0	
	Nitrofurantoin	0	0.0	0	0.0	1	1.2	0	0.0	
	Amoxicillin	1	3.2	4	4.9	1	1.2	0	0.0	
	Ampicillin	17	54.8	43	53.1	51	61.4	21	53.8	
	Trimethoprim/sulfamethoxazole	0	0.0	0	0.0	2	2.4	2	5.1	
		n=19	%	n=42	%	n=77	%	n=38	%	
Klebsiella pneumoniae										0.209
	Ciprofloxacin	0	0.0	0	0.0	0	0.0	1	2.6	
	Ceftriaxone	2	10.5	13	31.0	18	23.4	9	23.7	
	Piperacillin/tazobactam	0	0.0	1	2.4	0	0.0	0	0.0	
	Amoxicillin-clavulanate	0	0.0	0	0.0	2	2.6	3	7.9	
	Nitrofurantoin	0	0.0	1	2.4	0	0.0	0	0.0	
	Amoxicillin	1	5.3	0	0.0	1	1.3	1	2.6	
	Ampicillin	16	84.2	27	64.3	56	72.7	24	63.2	
		n=5	%	n=4	%	n=2	%	n=3	%	
Enterococcus faecalis										0.412
	Ciprofloxacin	3	60.0	0	0.0	1	50.0	0	0.0	
	Vancomycin	0	0.0	1	25.0	0	0.0	0	0.0	
	Erythromycin	0	0.0	1	25.0	0	0.0	1	33.3	
	Gentamicin	1	20.0	0	0.0	0	0.0	0	0.0	
	Nitrofurantoin	0	0.0	0	0.0	0	0.0	1	33.3	
	Clindamycin	1	20.0	1	25.0	0	0.0	0	0.0	
	Ampicillin	0	0.0	1	25.0	1	50.0	1	33.3	
		n=4	%	n=5	%	n=2	%	n=2	%	
Pseudomonas aeruginosa										0.911
	Ciprofloxacin	1	25.0	0	0.0	0	0.0	1	50.0	
	Piperacillin/tazobactam	2	50.0	3	60.0	1	50.0	1	50.0	
	Cefazolin	0	0.0	0	0.0	1	50.0	0	0.0	
	Ampicillin/Sulbactam	1	25.0	1	20.0	0	0.0	0	0.0	
	Trimethoprim/sulfamethoxazole	0	0.0	1	20.0	0	0.0	0	0.0	
		n=3	%	n=3	%	n=4	%	n=3	%	
Proteus mirabilis										0.666
	Ciprofloxacin	1	33.3	0	0.0	2	50.0	0	0.0	
	Nitrofurantoin	0	0.0	1	33.3	0	0.0	1	33.3	
	Ampicillin	1	33.3	2	66.7	2	50.0	2	66.7	
	Trimethoprim/sulfamethoxazole	1	33.3	0	0.0	0	0.0	0	0.0	

**Table 6:** Urine isolated bacterial resistance rate.

Urinary tract infection bacteria (resistant)			
Escherichia coli		n	%
	Ampicillin	132	56.4
	Ceftriaxone	79	33.8
	Ciprofloxacin	9	3.8
	Amoxicillin	6	2.6
	Trimethoprim/sulfamethoxazol	4	1.7
	Piperacillin/tazobactam	1	.4
	Amoxicillin/clavulanate	1	.4
	Cefazolin	1	.4
	Nitrofurantoin	1	.4
	Total	234	100.0
Klebsiella pneumoniae		n	%
	Ampicillin	123	69.9
	Ceftriaxone	42	23.9
	Amoxicillin/clavulanate	5	2.8
	Amoxicillin	3	1.7
	Ciprofloxacin	1	.6
	Piperacillin/tazobactam	1	.6
	Nitrofurantoin	1	.6
	Total	176	100.0
Enterococcus faecalis		n	%
	Ciprofloxacin	4	28.6
	Ampicillin	3	21.4
	Erythromycin	2	14.3
	Clindamycin	2	14.3
	Vancomycin	1	7.1
	Gentamicin	1	7.1
	Nitrofurantoin	1	7.1
	Total	14	100.0
Pseudomonas aeruginosa		n	%
	Piperacillin/tazobactam	7	53.8
	Ciprofloxacin	2	15.4
	Ampicillin/sulbactam	2	15.4
	Cefazolin	1	7.7
	Trimethoprim/sulfamethoxazol	1	7.7
	Total	13	100.0
Proteus mirabilis		n	%
	Ampicillin	7	53.8
	Ciprofloxacin	3	23.1
	Nitrofurantoin	2	15.4
	Trimethoprim/sulfamethoxazol	1	7.7
	Total	13	100.0
Acinetobacter baumannii		n	%
	Piperacillin/tazobactam	9	90.0
	Ampicillin	1	10.0
	Total	10	100.0
Citrobacter koseri		n	%
	Piperacillin/tazobactam	2	33.3
	Amoxicillin/clavulanate	2	33.3
	Ciprofloxacin	1	16.7
	Cefazolin	1	16.7
	Total	6	100.0
Enterobacter cloacae		n	%
	Amoxicillin/clavulanate	5	100.0
Enterococcus faecium		n	%
	Ampicillin	4	80.0
	Nitrofurantoin	1	20.0
	Total	5	100.0
Providencia stuartii		n	%
	Ampicillin	3	60.0
	Ceftriaxone	2	40.0
	Total	5	100.0
Enterobacter aerogenes		n	%
	Amoxicillin/clavulanate	3	75.0
	Amoxicillin	1	25.0
	Total	4	100.0

Regarding the isolates from respiratory sources, the most frequently isolated pathogen was* P. aeruginosa* (28.5%), followed by *K. pneumoniae *(17.6%), *A. baunmannii* (15.1%), MRSA (7.2%), and *E. coli* (5.6%) (Table 7).

**Table 7:** Bacteria isolated from respiratory specimen sources.

Bacteria		n=568	%
	Pseudomonas aeruginosa	162	28.5
	Klebsiella pneumoniae	100	17.6
	Acinetobacter baumannii	86	15.1
	Methicillin-resistant *Staphylococcus aureus*	41	7.2
	Escherichia coli	32	5.6
	Staphylococcus aureus	31	5.5
	Haemophilus influenzae	22	3.9
	Streptococcus pneumoniae	20	3.5
	Serratia marcescens	15	2.6
	Stenotrophomonas maltophilia	14	2.5
	Proteus mirabilis	11	1.9
	Burkholderia cepacia	5	.9
	Enterobacter cloacae	4	.7
	Klebsiella oxytoca	4	.7
	Enterobacter aerogenes	3	.5
	Citrobacter koseri	2	.4
	Providencia stuartii	2	.4
	Elizabethkingia meningoseptica	2	.4
	Alcaligenes faecalis	2	.4
	Serratia liquefaciens	1	.2
	Bacillus anthracis	1	.2
	Moraxella catarrhalis	1	.2
	Morganella morganii	1	.2
	Citrobacter freundii	1	.2
	Staphylococcus capitis	1	.2
	Providencia rettgeri	1	.2
	Achromobacter xylosoxidans	1	.2
	Cronobacter sakazakii	1	.2
	Haemophilus parainfluenzae	1	.2

Regarding the 162 respiratory *P. aeruginosa* isolates, most (51.9%) were resistant to piperacillin/tazobactam, followed by ciprofloxacin (25%), ampicillin (10.6%), ampicillin/sulbactam (3.8%), and meropenem (2.9%; p < 0.001). Meanwhile, *K. pneumoniae* was most resistant to ampicillin (82.7%), followed by ceftriaxone (9.2%), piperacillin/tazobactam (7.1%), and amoxicillin (1%; p = 0.153). Finally, the *A. baunmannii* isolates were most resistant to piperacillin/tazobactam (52.6%; p = 0.520). Table 8 and Table 9 give details of respiratory infection bacterial resistence. 

**Table 8:** Respiratory tract bacterial resistance rate.

Respiratory infection bacteria (Resistant)			
Pseudomonas aeruginosa		n	%
	Piperacillin/tazobactam	54	51.9
	Ciprofloxacin	26	25.0
	Ampicillin	11	10.6
	Ampicillin/sulbactam	4	3.8
	Meropenem	3	2.9
	Imipenem	2	1.9
	Ceftazidim	2	1.9
	Cefepime	1	1.0
	Tigecycline	1	1.0
	Total	104	100.0
Klebsiella pneumoniae		n	%
	Ampicillin	81	82.7
	Ceftriaxone	9	9.2
	Piperacillin/tazobactam	7	7.1
	Amoxicillin	1	1.0
	Total	98	100.0
Acinetobacter baumannii		n	%
	Piperacillin/tazobactam	41	52.6
	Ampicillin	30	38.5
	Ciprofloxacin	4	5.1
	Meropenem	1	1.3
	Ceftriaxone	1	1.3
	Amoxicillin/clavulanate	1	1.3
	Total	78	100.0
Methicillin-resistant *Staphylococcus aureus*		n	%
	Cefazolin	8	72.7
	Clindamycin	2	18.2
	Piperacillin/tazobactam	1	9.1
	Total	11	100.0
Escherichia coli		n	%
	Ampicillin	16	59.3
	Ceftriaxone	10	37.0
	Amoxicillin	1	3.7
	Total	27	100.0
Staphylococcus aureus		n	%
	Clindamycin	4	40.0
	Erythromycin	3	30.0
	Trimethoprim/sulfamethoxazol	2	20.0
	Cefazolin	1	10.0
	Total	10	100.0
Haemophilus influenzae		n	%
	Ciprofloxacin	1	50.0
	Ampicillin	1	50.0
	Total	2	100.0
Streptococcus pneumoniae		n	%
	Ceftriaxone	2	20.0
	Erythromycin	2	20.0
	Clindamycin	2	20.0
	Penicillin	2	20.0
	Vancomycin	1	10.0
	Levofloxacin	1	10.0
	Total	10	100.0
Serratia marcescens		n	%
	Amoxicillin/clavulanate	10	76.9
	Ciprofloxacin	1	7.7
	Cefazolin	1	7.7
	Ceftazidim	1	7.7
	Total	13	100.0
Stenotrophomonas maltophilia		n	%
	Trimethoprim/sulfamethoxazol	2	40.0
	Ciprofloxacin	1	20.0
	Piperacillin/tazobactam	1	20.0
	Levofloxacin	1	20.0
	Total	5	100.0

**Table 9:** Resistance rate changes over the study period among respiratory tract bacteria.

	Respiratory infection bacteria (Resistant)	Year of diagnosis								p-value
		2016		2017		2018		2019		
		n=25	%	n=35	%	n=26	%	n=18	%	
Pseudomonas aeruginosa										<0.001
	Meropenem	2	8.0	1	2.9	0	0.0	0	0.0	
	Ciprofloxacin	2	8.0	2	5.7	16	61.5	6	33.3	
	Piperacillin/tazobactam	8	32.0	27	77.1	9	34.6	10	55.6	
	Imipenem	0	0.0	1	2.9	1	3.8	0	0.0	
	Ceftazidim	2	8.0	0	0.0	0	0.0	0	0.0	
	Cefepime	0	0.0	0	0.0	0	0.0	1	5.6	
	Tigecycline	0	0.0	0	0.0	0	0.0	1	5.6	
	Ampicillin/sulbactam	1	4.0	3	8.6	0	0.0	0	0.0	
	Ampicillin	10	40.0	1	2.9	0	0.0	0	0.0	
		n=8	%	n=31	%	n=28	%	n=31	%	
Klebsiella pneumoniae										0.153
	Ceftriaxone	1	12.5	3	9.7	2	7.1	3	9.7	
	Piperacillin/tazobactam	0	0.0	6	19.4	1	3.6	0	0.0	
	Amoxicillin	0	0.0	0	0.0	0	0.0	1	3.2	
	Ampicillin	7	87.5	22	71.0	25	89.3	27	87.1	
		n=22	%	n=35	%	n=10	%	n=11	%	
Acinetobacter baumannii										0.520
	Meropenem	0	0.0	1	2.9	0	0.0	0	0.0	
	Ciprofloxacin	2	9.1	1	2.9	1	10.0	0	0.0	
	Ceftriaxone	0	0.0	1	2.9	0	0.0	0	0.0	
	Piperacillin/tazobactam	9	40.9	19	54.3	5	50.0	8	72.7	
	Amoxicillin/clavulanate	0	0.0	0	0.0	0	0.0	1	9.1	
	Ampicillin	11	50.0	13	37.1	4	40.0	2	18.2	
		n=1	%	n=2	%	n=6	%	n=2	%	
Methicillin-resistant *Staphylococcus aureus*										0.636
	Piperacillin/tazobactam	0	0.0	0	0.0	1	16.7	0	0.0	
	Cefazolin	0	0.0	2	100.0	4	66.7	2	100.0	
	Clindamycin	1	100.0	0	0.0	1	16.7	0	0.0	
		n=4	%	n=7	%	n=11	%	n=5	%	
Escherichia coli										0.890
	Ceftriaxone	1	25.0	2	28.6	5	45.5	2	40.0	
	Amoxicillin	0	0.0	1	14.3	0	0.0	0	0.0	
	Ampicillin	3	75.0	4	57.1	6	54.5	3	60.0	

## Discussion

The growing incidence of antibiotic resistance is a substantial concern globally and is considered the main hurdle to the effectiveness of treating bacterial infection/ Table 10 shows the list of antibiotic resistance over time. Importantly, the resistance discovery dates in Table 10 are according to the observations during clinical practice, however, the resistance might have appeared earlier based on the findings from laboratory-based experiments.

**Table 10:** Selected germs showing resistance over time.

Antibiotic Approved or Released	Year Released	Resistant Germ Identified	Year Identified
Penicillin	1943	Penicillin-resistant *Streptococcus pneumoniae*; Penicillinase-producing *Neisseria gonorrhoeae*	1967, 1976 [21]
Vancomycin	1958	Plasmid-mediated vancomycin-resistant *Enterococcus faecium*; Vancomycin-resistant *Staphylococcus aureus*	1988 [22], 2002 [23]
Amphotericin B	1959	Amphotericin B-resistant *Candida auris*	2016 [24]
Methicillin	1960	Methicillin-resistant *Staphylococcus aureus*	1960 [25]
Extended-spectrum cephalosporins	1980 (Cefotaxime)	Extended-spectrum beta-lactamase- producing *Escherichia coli*	1983 [26]
Azithromycin	1980	Azithromycin-resistant *Neisseria gonorrhoeae*	2011 [27]
Imipenem	1985	*Klebsiella pneumoniae carbapenemase* (KPC)-producing *Klebsiella pneumoniae*	1996 [28]
Ciprofloxacin	1987	Ciprofloxacin-resistant *Neisseria gonorrhoeae*	2007 [29]
Fluconazole	1990 (FDA approved)	Fluconazole-resistant *Candida*	1988 [30]
Caspofungin	2001	Caspofungin-resistant *Candida*	2004 [31]
Daptomycin	2003	Daptomycin-resistant methicillin-resistant *Staphylococcus aureus*	2004 [32]
Ceftazidime-avibactam	2015	Ceftazidime-avibactam-resistant KPC-producing *Klebsiella pneumoniae*	2015 [33]

The growing prevalence of ABR bacteria may affect the capability to control infectious diseases by reducing treatment effectiveness, prolonging illness duration, raising mortality rates, and increasing healthcare costs. This study aimed to identify the common rate of bacterial resistance against antibacterial agents in MNGHA, Jeddah, Saudi Arabia, and to assess the practice of appropriate antibiotic treatment.

In this study, the top 10 most common causative agents of UTIs and RTIs were *E. coli *(26.4%), *K. pneumonia* (24.2%), *P. aeruginosa* (16.9%), *A. baumannii* (8.4%), MRSA (3.6%), *E. faecalis* (3%), *S. aureus* (2.9%), *P. mirabilis* (2.1%), *H. influenzae* (2%), and *S. pneumoniae* (1.8%). Despite the lack of significant differences between isolated organisms across age groups, most of the causative organisms identified in this study were more prevalent (25.5%) in the group aged 76-85 years than in other age groups.

The most common causative agent of UTIs in this study was E. coli (46.7%), followed by *K. pneumoniae* (30.5%), *E. faecalis* (6%), *P. aeruginosa* (5.5%), and *P. mirabilis* (2.2%). These findings are consistent with local and global epidemiological data. In 2012, the most frequently identified bacteria in urinary isolates from female outpatients in the United States was E. coli (64.9%), followed by *K. pneumonia* (10.1%), *P. mirabilis *(5%), *E. faecalis* (4.1%), and *P. aeruginosa* (2.7%) [21]. In 2018, the most common microbial causative agent of UTIs in isolates collected from major tertiary hospitals in Riyadh, Saudi Arabia, was *E. coli *(52%), followed by *K. pneumoniae* (15%), *P. aeruginosa* (8%), *S. agalactiae* (7%), and *E. faecalis* (5%) [22].

Among the most commonly prescribed antibiotics for UTI management, the most commonly identified uropathogen in the present study (*E. coli*) was most resistant to ampicillin (56.4%), followed by ceftriaxone (33.8%), ciprofloxacin (3.8%), amoxicillin (2.6%), and sulfamethoxazole/trimethoprim (1.7%). A similar result was reported in a study of three governmental hospitals (Najran General Hospital, Khalid Hospital, and Najran University Hospital) in the Najran region of Saudi Arabia, which aimed to investigate the antimicrobial resistance patterns of 136 outpatient urine samples. In this prior study, *E. coli* (58.5%) was the most common causative agent of UTIs, with an ampicillin resistance rate of 56.94% [23]. Although the emergence of antibiotic resistance may vary regionally and geographically, the present results regarding UTIs appear to be consistent with global antibiotic resistance data. In a multinational, multicenter study of 19,756 urine samples collected from 2003 to 2010, *E. coli *showed aminopenicillin antibiotic resistance rates of 42% in Northern Europe, 59% in Southern Europe, 60% in Asia, and 53% in South America and Africa [24].

Among the isolates from respiratory sources, the most common bacterial pathogen was *P. aeruginosa*, totaling 162 isolates (28.5%). Of these *P. aeruginosa* isolates, most (51.9%) were resistant to piperacillin/tazobactam, followed by ciprofloxacin (25%), ampicillin (10.6%), ampicillin/sulbactam (3.8%), and meropenem (2.9%). Similarly, in a study of 10 medical centers from all regions of Canada, the P. aeruginosa isolate was the predominant respiratory organism (26.2%; 423/1,612 isolates) [25]. These findings are also consistent with those of a more recent study in which more than 20% of *P. aeruginosa* isolates were resistant to piperacillin/tazobactam [26]. In the present study, *K. pneumoniae* was the second-most prevalent bacteria (17.6%) in the isolates from respiratory sources. Regarding the 100 *K. pneumoniae *isolates, ampicillin resistance was highest (82.7%), followed by ceftriaxone (9.2%) and piperacillin/tazobactam (7.1%) resistance. This is contrary to the study by Al-Zalabani et al., in which the overall resistance found in 11,507 *K.pneumoniae* isolates was 61.7%, with remarkably high resistance rates of 80.4% and 58.7% to piperacillin and piperacillin/tazobactam, respectively [34]. There have been varied reports on the prevalence of *Klebsiella* species in different parts of the world [25].

The present study revealed high resistance rates for commonly used antibiotics, including ampicillin, amoxicillin/clavulanic acid, and sulfamethoxazole/trimethoprim. It has been suggested that the high rate of resistance to first-line therapies is due to several factors, including antibiotic misuse or self-medication and the ongoing unlawful dispensing of antibiotics in community pharmacies [27], despite the regulations and efforts applied by the Saudi Ministry of Health to alleviate emerging antibiotic resistance. The prescription of antibiotics in dentistry is of particular concern, as it has been reported that general dental practitioners are lacking knowledge regarding prescription of antibiotics in endodontic treatment and situations requiring prophylactic antibiotics [28]. Further investigation is needed to assess these concerns.

Importantly, an initiative strategy by the CDC known as the Antimicrobial Stewardship Programs (ASPs), which was developed as a preventive measure for increased resistance, outlined a set of seven integrated elements to be used by medical care providers globally: leadership commitment, accountability, pharmacy expertise, action, tracking, reporting, and education [35]. Thus, the Saudi Ministry of Health has put efforts into implementing the ASPs in healthcare facilities [36].

Limitations

The population in our study is limited to one particular tertiary healthcare center in Jeddah, Saudi Arabia and this may not represent the antimicrobial resistance trends in another region within the same country. Additionally, the present study was based on information from patients’ files. The BESTCare patient data documentation system, which enabled physicians to monitor each patient’s course of antibiotics and guide them through proper and reliable antibiotic management, helped the researchers track patient records. However, incomplete documentation of certain prescribed antibiotics and their impacts on patient outcomes was considered a limitation. Also, communication with infectious disease specialists could have helped in understanding the hospital’s protocol regarding antibiotic resistance.

## Conclusions

In this study, high levels of antibiotic resistance were observed in both Gram-negative and Gram-positive bacteria. For better implementation of antibacterial stewardship and the optimization of empirical therapies, updated epidemiological data from a given community is necessary to determine the actual pattern of bacterial resistance within that community. Moreover, future studies should assess the impact of maternal antibiotic administration on newborns and the spread of ABR bacteria.
